# Clinical and Molecular Characterization of *TCIRG1*-Related Autosomal Recessive Osteopetrosis with Current Therapeutic Approaches

**DOI:** 10.3390/biomedicines14050958

**Published:** 2026-04-22

**Authors:** Sabina E. Nagieva, Svetlana A. Smirnikhina

**Affiliations:** Research Centre for Medical Genetics, 115522 Moscow, Russia

**Keywords:** *TCIRG1*, infantile malignant osteopetrosis, osteoclasts, allogeneic hematopoietic stem cell transplantation, lentiviral vectors

## Abstract

**Background/Objectives:** *TCIRG1*-associated infantile osteopetrosis is a severe hereditary disorder caused by impaired osteoclast function, leading to osteosclerosis, hematological abnormalities, neurological complications, and early mortality. Early diagnosis and intervention are critical. **Methods:** A literature-based analysis was performed on clinical manifestations, outcomes of allogeneic hematopoietic stem cell transplantation (HSCT), immunomodulatory therapy, and experimental gene therapy and cell-based approaches, including lentiviral vectors and patient-derived induced pluripotent stem cells (iPSCs). **Results:** Allogeneic HSCT is the only established curative therapy, restoring osteoclast function and preventing severe complications. Early transplantation with HLA-matched donors and myeloablative conditioning provides optimal outcomes. Interferon γ1b can transiently enhance osteoclast activity but is not curative and shows variable efficacy. Preclinical studies demonstrate that lentiviral *TCIRG1* delivery and transgenic correction in patient-derived iPSCs restore osteoclast function and bone resorption, with stable gene expression and minimal toxicity. Base and prime editing approaches offer potential for precise correction of single-nucleotide *TCIRG1* variants, minimizing risks associated with double-strand DNA breaks. **Conclusions:** Allogeneic HSCT remains the standard therapy for *TCIRG1*-associated infantile osteopetrosis. Gene therapy and cell-based strategies represent promising adjuncts or alternatives, potentially avoiding immune-related complications and expanding therapeutic options. Further studies are needed to ensure safety, stable engraftment, and long-term efficacy, supporting translation of gene therapy into clinical practice.

## 1. Introduction

Osteopetrosis encompasses a group of disorders characterized by increased bone density accompanied by pathological fragility. These alterations are associated with pathogenic variants in specific genes responsible for the regulation of osteoclast-mediated bone resorption. Currently, osteopetrosis has been described in forms with autosomal-dominant, autosomal-recessive, and X-linked inheritance types [1].

In addition to genetic heterogeneity, this group of disorders exhibits significant clinical variability. Phenotypic manifestations in patients with osteopetrosis can range from asymptomatic or mild forms to severe presentations associated with early infantile lethality [2].

**Figure 1:**
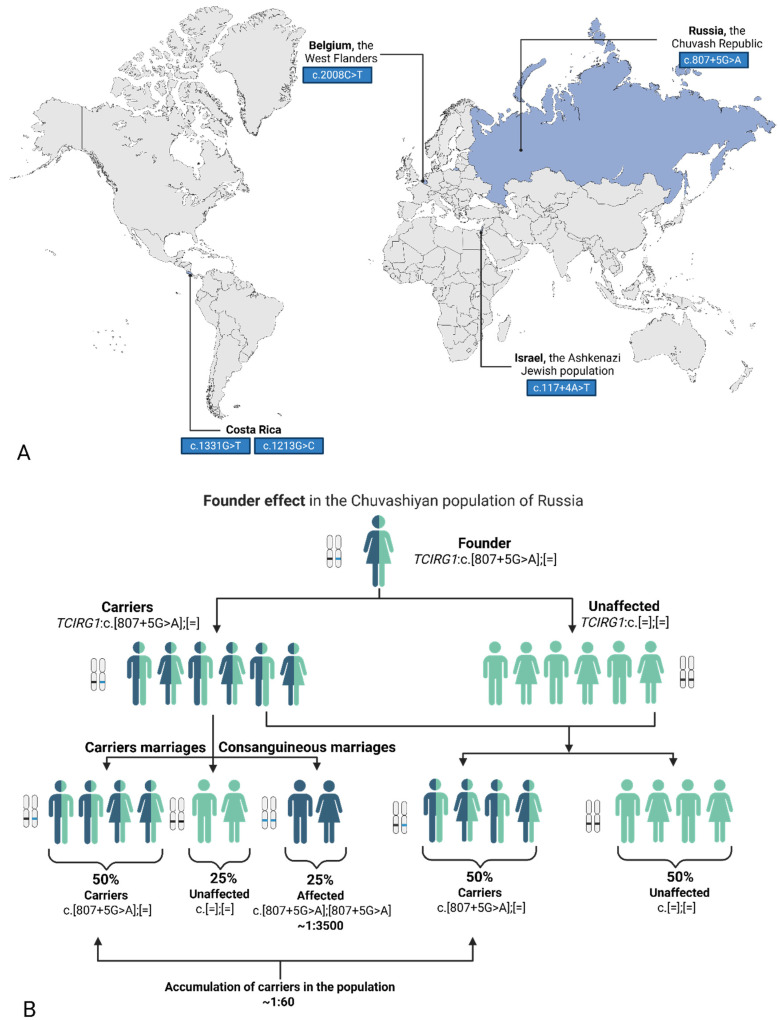
(**A**) World map with common variants in certain populations [3,4,5,6]. (**B**) Schematic representation of the founder effect and the accumulation of a variant in the population. Ilustrated by the common variant *TCIRG1*: c.807+5G>A in the Chuvashiyan population of Russia. Image created using BioRender.com. Nagieva, S. (2026). (**A**) https://app.biorender.com/illustrations/6999ae139f8ceaf0cf4127dd. (**B**) https://app.biorender.com/illustrations/699d69878ee77b82fb1f1866 (all accessed on 28 February 2026).

Early-onset autosomal-recessive forms of osteopetrosis are considered the most clinically severe. Approximately 50–60% of all autosomal-recessive cases are attributed to *TCIRG1*-associated osteopetrosis [3]. The high frequency of pathogenic variants in the *TCIRG1* gene may be influenced by several population-genetic factors. Several studies have reported the presence of a founder effect, leading to an increased prevalence of specific *TCIRG1* variants in isolated or ethnically homogeneous populations. For example, the c.807+5G>A variant in the Chuvash population of Russia, p.Arg670Ter in the population of West Flanders, Belgium, p.Arg444Leu and p.Gly405Arg in individuals of Costa Rican descent, as well as the c.117+4A>T variant in the Ashkenazi Jewish population [3,4,5,6] (Figure 1A). Furthermore, the autosomal-recessive mode of inheritance contributes to the maintenance and accumulation of mutant alleles in the population, thereby increasing the overall contribution of this gene to the disease burden of osteopetrosis (Figure 1B).

The clinical course of *TCIRG1*-associated osteopetrosis generally corresponds to the infantile (malignant) form of the disease and is characterized by rapid progression, with onset during the first months of life [7]. Despite supportive therapy, without timely intervention, the disease often leads to early childhood mortality.

*TCIRG1*-associated osteopetrosis exhibits high genetic heterogeneity, with more than 120 different pathogenic variants described, including missense, nonsense, splice-site changes, small insertions/deletions, and large deletions, underscoring genetic complexity [1].

The severity of *TCIRG1*-associated osteopetrosis limits available therapeutic options. Currently, allogeneic hematopoietic stem cell transplantation (HSCT) is considered the primary treatment, capable of partially restoring osteoclast function. However, the efficacy of this approach largely depends on the timing of transplantation and the patient’s clinical condition at the time of the procedure. Additionally, the procedure carries a risk of severe complications and does not always prevent already established neurological damage.

In this context, gene- and cell-based replacement therapies, as well as various genome editing strategies, are of particular interest, offering the prospect of targeted correction of pathogenic *TCIRG1* variants. The aim of the present review is to systematically summarize current knowledge on the pathogenesis of the disease and to analyze prospective therapeutic approaches.

## 2. Methods

A structured literature search was conducted in the electronic database PubMed and in authoritative clinical genetic resources, including GeneReviews (NCBI Bookshelf), to identify relevant publications. The search covered studies published from January 1992 to December 2025. Additional relevant articles were identified through manual screening of reference lists. The search strategy combined Medical Subject Headings (MeSH) terms and free-text keywords, including: “*TCIRG1*”, “autosomal recessive osteopetrosis”, “infantile malignant osteopetrosis”, “osteoclast dysfunction”, “hematopoietic stem cell transplantation”, “HSCT”, “gene therapy”, “lentiviral vector”, “induced pluripotent stem cells”. Inclusion criteria comprised: original research articles, clinical studies, preclinical studies, and relevant translational research, studies specifically addressing *TCIRG1*-related osteopetrosis or closely related osteoclast disorders, and publications in English. Both human and experimental model studies were considered where relevant to therapeutic mechanisms. Exclusion criteria included: case reports lacking therapeutic or mechanistic relevance, studies not specifically addressing *TCIRG1* or osteoclast function. Data extraction focused on clinical outcomes, therapeutic efficacy, safety profiles, and mechanistic insights related to osteoclast function restoration. The collected data were qualitatively synthesized to compare established and emerging therapeutic strategies.

## 3. Molecular Mechanisms and Clinical Manifestations of *TCIRG1*-Associated Osteopetrosis

### 3.1. Function of the TCIRG1 Gene and the Role of V-ATPase in Osteoclast-Mediated Bone Resorption

**Figure 2:**
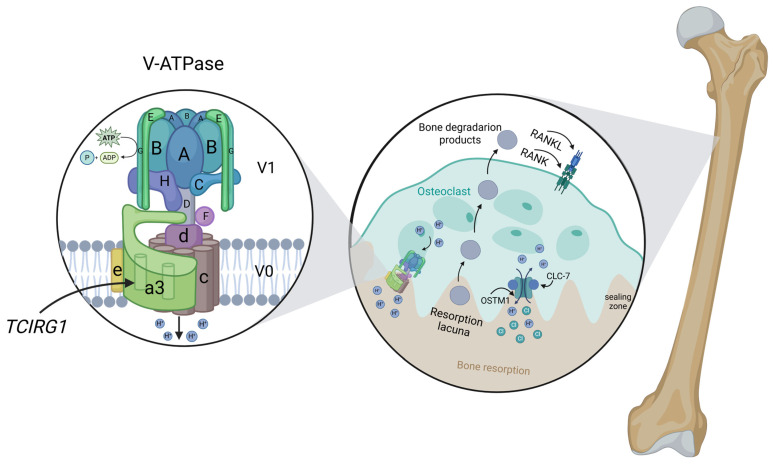
Structure and function of V-ATPase in osteoclast-mediated bone resorption. The V1 and V0 domains of the proton pump are shown, highlighting the a3 subunit encoded by *TCIRG1*. In osteoclasts, V-ATPase actively transports protons into the resorption lacuna, thereby acidifying the extracellular compartment and creating the low-pH environment required for bone matrix degradation. The CLC-7/OSTM1 complex acts as a chloride-proton exchanger that maintains electrochemical neutrality by facilitating chloride ion movement in coordination with proton pumping. This charge compensation prevents electrical buildup across the ruffled border membrane, which would otherwise limit V-ATPase activity and impair sustained acidification. Accordingly, the CLC-7/OSTM1 complex is essential for efficient mineral dissolution downstream of RANK–RANKL signaling and for optimal activity of lysosomal enzymes, including cathepsin K, within the resorption lacuna [8,9,10,11]. Image created using BioRender.com. Nagieva, S. (2026). https://app.biorender.com/illustrations/69a34d0d920a049894f22039 (accessed on 5 March 2026).

The *TCIRG1* gene, located on chromosome 11, plays a critical role in osteoclast-mediated bone resorption as well as in T-lymphocyte activation and immune response. This gene encodes two main protein isoforms: “a” and “b” (Figure 2).

Isoform “a”, also known as OC116, is encoded by the full-length transcript of *TCIRG1*, consisting of 20 exons, and represents the a3 subunit of the transmembrane domain of vacuolar H^+^-ATPase (V-ATPase). This protein is expressed in osteoclasts and constitutes a key component of the proton pump, which mediates the transport of hydrogen ions into the resorption lacuna. Proton transport regulates the pH of both the osteoclast and its microenvironment, which is essential for intracellular processes such as protein sorting, activation of zymogens (proenzymes) for mineral matrix degradation, and the maintenance of normal bone resorption [8,9].

When the proton pump is dysfunctional, osteoclasts accumulate but remain functionally inactive, failing to effectively resorb bone tissue. This leads to impaired bone remodeling, where old bone is not removed and new bone is formed with defective mineralization. Consequently, bones become pathologically dense yet fragile, with compromised microarchitecture, increasing the risk of deformities and fractures.

Moreover, impaired osteoclastic resorption affects the bone microenvironment, including extramedullary hematopoiesis abnormalities, reduced mineralization, and dysregulation of signaling pathways involved in bone growth, such as RANK/RANKL/OPG. These molecular mechanisms collectively contribute to the clinical manifestations of osteopetrosis, including skeletal deformities, anemia, and neurological complications [10].

Isoform “b”, also known as *TIRC7*, represents a truncated variant generated through alternative splicing. This transcript originates from exon 5 of the *TCIRG1* gene and encodes a protein lacking certain domains required for proper membrane integration and full functionality of the vacuolar H^+^-ATPase [9].

Isoform “b” is predominantly expressed in immune system cells and is thought to participate in the regulation of intracellular processes not directly related to bone resorption. The TIRC7 protein plays a key role in modulating the immune response and suppressing excessive T-lymphocyte activation [12]. Its modulation reduces T-cell proliferation and the secretion of proinflammatory cytokines, such as IFN-γ, thereby helping to prevent aggressive immune reactions against transplanted tissue. In both clinical and experimental models, increased TIRC7 expression in the graft is associated with a reduced risk of acute rejection, making this protein a promising target for immunotherapeutic strategies aimed at supporting graft engraftment [13,14].

### 3.2. Major Clinical Forms of TCIRG1-Associated Osteopetrosis

**Severe infantile (malignant) *TCIRG1*-associated osteopetrosis with autosomal-recessive inheritance** is characterized by severe manifestations with onset in the first months of life and often leads to early childhood mortality due to the lack of effective treatment. This form is most commonly associated with biallelic loss-of-function variants (including nonsense, frameshift, and canonical splice-site variants), which result in complete or near-complete loss of TCIRG1 protein function and impaired osteoclast-mediated bone resorption. Without timely treatment, the disease often leads to early childhood mortality, primarily due to a combination of bone marrow failure, severe infections, and neurological complications [7].

Due to the clinical heterogeneity of the disease, some patients exhibit a **milder form of *TCIRG1*-associated osteopetrosis**. This form is typically associated with variants allowing partial preservation of protein function, such as certain missense or non-canonical splice-site mutations. These patients may present with later disease onset, relatively normal growth, and absence of severe hematological or neurological abnormalities. Functional studies have demonstrated that some of these variants lead to aberrant splicing, including exon skipping or intron retention, while still permitting the production of a small proportion of normal transcripts. This residual expression enables partial V-ATPase activity, which is sufficient to maintain limited osteoclast function and results in a less severe clinical phenotype [15].

The literature reports recently described cases of **autosomal dominant *TCIRG1*-associated osteopetrosis.** This form is typically linked to heterozygous missense variants with a dominant-negative effect, leading to partial impairment of proton pump function. Clinically, these patients tend to exhibit a milder phenotype, with disease manifestation occurring in childhood or even adulthood [16,17].

Overall, emerging data suggest a correlation between the type and functional impact of *TCIRG1* variants and disease severity. Variants causing complete loss of protein function are generally associated with severe, early-onset disease, whereas variants preserving partial activity tend to result in milder or delayed phenotypes. However, genotype–phenotype correlations remain incomplete, as there is significant heterogeneity among patients with similar variants. This variability may reflect the influence of modifier genes, epigenetic factors, or differences in residual protein activity, highlighting the need for integrated clinical and molecular assessment in each case.

### 3.3. Key Clinical Manifestations of Autosomal Recessive TCIRG1-Associated Osteopetrosis

**Figure 3:**
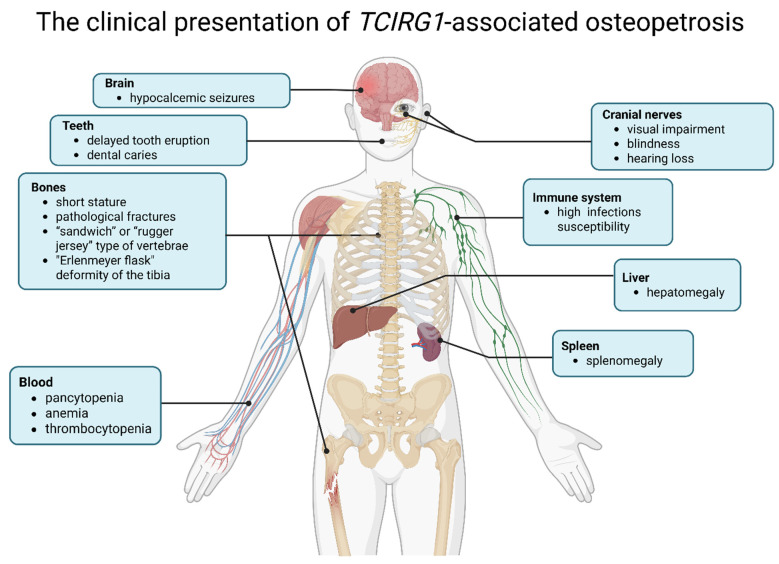
Clinical manifestations of autosomal recessive *TCIRG1*-associated osteopetrosis [7]. Image created using BioRender.com. Nagieva, S. (2026). https://app.biorender.com/illustrations/69a36f52268569fe8a3a58a4 (accessed on 6 March 2026).

The clinical presentation of *TCIRG1*-associated osteopetrosis comprises several major groups of manifestations (Figure 3) [7]:

- Skeletal manifestations: generalized osteosclerosis, increased bone density accompanied by bone fragility, pathological fractures, skeletal deformities, as well as delayed tooth eruption and dental caries. Diagnostic radiographic features include Erlenmeyer flask deformity of the tibia, metaphyseal widening of the tibia and femur, and flattened vertebrae of the “sandwich” or “rugger jersey” type. Increased bone density is characterized by a funnel-shaped configuration of the metaphyses and typical alternating radiolucent bands that reflect differences in the mineral composition of bone tissue.
- Hematological abnormalities: pancytopenia resulting from narrowing of the bone marrow cavities, anemia, thrombocytopenia, and hepatosplenomegaly due to extramedullary hematopoiesis.
- Neurological manifestations: compression of cranial nerves, particularly the optic and auditory nerves, leading to visual impairment, blindness, and hearing loss, seizures associated with hypocalcemia may also occur.
- Immunological abnormalities: increased susceptibility to infections associated with impaired bone marrow hematopoiesis and dysregulated immune responses.

The disease typically manifests during the first weeks or months of life. Initial clinical suspicion usually arises from a combination of signs, including anemia, thrombocytopenia, hepatosplenomegaly, visual impairment, seizures due to hypocalcemia, and characteristic skeletal changes on radiographic imaging, as described earlier [18]. Although the clinical picture often becomes apparent by 1–3 months of age, diagnosis may be delayed because of the rarity of the condition (approximately 1:200,000–300,000 newborns, excluding regions with founder effect variants) and nonspecific early symptoms, frequently resembling other hematologic or metabolic disorders. An additional challenge is the need for genetic testing, which may take several weeks and is essential for determining eligibility for curative treatment. Once the diagnosis is confirmed, the search for a donor for hematopoietic stem cell transplantation—the only curative therapy—must begin. This process can also be time-consuming because of the need to identify an HLA-compatible donor [19]. Therefore, even when the disease is recognized relatively early, the main difficulties often involve not only timely diagnosis but also the subsequent identification of a suitable donor and preparation for transplantation.

For molecular confirmation, multigene panels that include *TCIRG1* and other osteopetrosis-associated genes are generally preferred due to their diagnostic precision and cost efficiency. When clinical suspicion strongly favors *TCIRG1*-related disease, single-gene testing with sequence analysis can detect missense, nonsense, and splice-site variants. If only one pathogenic variant is identified, complementary deletion/duplication analysis is recommended to exclude large rearrangements. In populations or geographic regions where a founder effect has been established and a recurrent pathogenic variant is prevalent, targeted testing for the specific variant may represent a rapid and cost-effective first-line strategy. Comprehensive genomic approaches, such as whole-exome or whole-genome sequencing, are particularly useful in atypical presentations or when the phenotype overlaps with other skeletal dysplasias, allowing broader differential diagnostic coverage [7].

### 3.4. Differential Diagnosis of TCIRG1-Associated Autosomal Recessive Osteopetrosis

*TCIRG1*-associated autosomal recessive osteopetrosis represents one of the most severe forms of infantile malignant osteopetrosis. Differential diagnosis must therefore be performed in a structured manner, as different genetic subtypes vary substantially in pathophysiology, clinical course, neurological prognosis, and eligibility for hematopoietic stem cell transplantation (HSCT).

Pathogenic variants in *CLCN7* may cause either autosomal recessive infantile osteopetrosis or a milder autosomal dominant form. In recessive cases, the clinical phenotype may resemble TCIRG1 deficiency, with severe hematological involvement and bone marrow failure. These patients are generally considered as potential candidates for HSCT, because transplantation can correct hematopoietic and osteoclast-related defects. However, neurological impairment may persist or be only partially reversible due to intrinsic neuronal involvement.

In contrast, *OSTM1*-associated osteopetrosis is characterized by a severe early-onset neurodegenerative phenotype. Although HSCT may restore hematopoietic and osteoclastic function, it does not prevent progressive neurological deterioration, which dominates the clinical course. Consequently, the overall benefit of HSCT in these patients is limited, and prognosis remains poor despite successful engraftment.

A fundamentally different therapeutic category is represented by osteopetrosis due to pathogenic variants in *TNFSF11* or *TNFRSF11A*, which disrupt the RANKL–RANK signaling pathway. These forms are characterized by an osteoclast-poor phenotype due to impaired osteoclast differentiation. As the primary defect lies upstream of osteoclast development, HSCT is generally not effective and not considered a curative option, which is a key point of distinction in clinical decision-making.

*TCIRG1*-associated disease is characterized by defective osteoclast acidification with preserved osteoclast precursors, making it highly responsive to HSCT, which remains the standard curative therapy. Therefore, precise molecular diagnosis is essential, as it directly determines HSCT eligibility, expected treatment response, and neurological prognosis [7].

Summarizing the data presented in this section, the diagnostic approach integrates clinical, radiological, laboratory, and genetic data and can be structured as follows (Table 1):

## 4. Therapeutic Approaches to the Treatment of *TCIRG1*-Associated Autosomal Recessive Osteopetrosis

### 4.1. Allogeneic Hematopoietic Stem Cell Transplantation (HSCT)

At present, allogeneic HSCT is the primary therapeutic approach for infantile *TCIRG1*-associated osteopetrosis.

The pathogenetic rationale for HSCT is based on the hematopoietic origin of osteoclasts from CD34^+^ stem cells. Following transplantation, recipient hematopoiesis is replaced by donor-derived cells, enabling differentiation into functionally competent osteoclasts and restoration of bone resorption. This directly corrects the primary cellular defect underlying *TCIRG1*-associated disease [20].

A key condition for the effectiveness of HSCT is that, in *TCIRG1*-osteopetrosis, the defect is limited to osteoclasts, in contrast to forms associated with impaired osteoblast maturation and activation (e.g., *RANKL*-associated osteopetrosis) [21] or with disturbances in the maintenance of neuronal homeostasis [22,23] (*OSTM1*-associated osteopetrosis with severe neurodegeneration), in which transplantation is ineffective.

According to the recommendations of the European Society for Blood and Marrow Transplantation (EBMT) and the European Society for Immunodeficiencies (ESID), the main indications for HSCT include a severe disease course with signs of bone marrow failure, hepatosplenomegaly, and compression of cranial nerves, particularly the optic nerve [24,25]. It has been demonstrated that the best outcomes are achieved with early transplantation—within the first months of life, preferably before 3–6 months of age—which allows prevention of irreversible neurological complications [25,26].

The choice of graft source is an important prognostic factor. The most favorable outcomes are observed when an HLA-matched related donor is used, whereas transplantation from an unrelated donor or the use of umbilical cord blood are considered alternative options in the absence of a compatible relative [24,25]. However, the use of umbilical cord blood is associated with slower engraftment and an increased risk of graft rejection despite its availability [26].

The conditioning regimen prior to allogeneic HSCT in infantile osteopetrosis is generally myeloablative in nature and aims at the complete replacement of the defective osteoclastic pool with donor-derived cells. Busulfan-based regimens (busulfan + fludarabine), which provide sufficient myeloablation and create a niche for graft engraftment, are most widely used. Treosulfan-based regimens in combination with fludarabine and thiotepa represent an alternative in high-risk groups [27,28].

The efficacy of allogeneic HSCT is largely determined by the adequacy of the conditioning regimen and the success of graft engraftment. The achievement of stable donor hematopoiesis and restoration of functional osteoclast activity constitute the key prerequisites for a clinical response to therapy.

An analysis of a cohort of 200 patients with *TCIRG1*-associated osteopetrosis reported an 88% five-year relapse-free survival among recipients of allogeneic hematopoietic stem cell transplantation from HLA-identical donors, while patients receiving HSCT from HLA-matched unrelated donors had a five-year progression-free survival of 80%. In contrast, data from the Center for International Blood and Marrow Transplant Research showed a 62% relapse-free survival rate for individuals transplanted with HLA-identical donor grafts. These discrepancies are primarily explained by:

- differences in median age at transplantation (earlier transplantation correlates with improved outcomes),
- variation in conditioning intensity and protocols,
- differences in donor availability and HLA matching stringency,
- heterogeneous inclusion of patients with advanced disease and pre-existing neurological damage,
- and differences in study design, registry structure, and follow-up duration [29].

More recently, a large EBMT cohort study including 746 pediatric patients (1990–2022) provided the most comprehensive dataset to date. *TCIRG1* variants accounted for approximately two-thirds of cases. This study reported a 3-year overall survival of 69%**,** improving over time from 50% in earlier eras to 77% in recent years, reflecting advances in donor selection, supportive care, and earlier transplantation. Event-free survival was approximately 61%**,** with graft failure occurring in ~21% of patients. Adverse prognostic factors included HLA mismatch, splenomegaly, poor performance status, and use of cord blood, while treatment in experienced centers significantly improved outcomes [30].

Among long-term survivors, up to 70% present with persistent visual impairment, while approximately 10% develop hearing impairment and delayed motor development [24,30]. These complications are largely attributable to irreversible pre-transplant neurological damage rather than transplant failure.

Allogeneic hematopoietic stem cell transplantation in *TCIRG1*-associated infantile osteopetrosis is associated with several significant limitations and complications. One of the principal factors is the risk of transplantation-related morbidity and mortality. Reported complication rates include:

- Graft failure: ~20–21% in large EBMT cohorts [25,30]
- Acute and chronic GVHD: approximately 15–30% depending on donor type and conditioning intensity [31]
- Transplant-related mortality: variable, ranging from ~10% in early transplantation to higher rates in late or high-risk cases
- Infectious complications: frequent in the early post-transplant period due to profound immunosuppression
- Veno-occlusive disease (VOD): reported in a minority of patients, particularly with busulfan-based conditioning
- Pulmonary complications (e.g., interstitial pneumonitis, pulmonary hypertension): rare but clinically significant
- Post-transplant hypercalcemia: observed in a subset of patients due to restoration of osteoclast function and increased bone resorption [32,33]

An important limitation of HSCT is its inability to reverse already established irreversible changes, particularly optic nerve damage and other neurological impairments, underscoring the need for early therapeutic intervention.

HSCT remains a complex and potentially high-risk treatment modality that requires careful patient selection and optimization of treatment protocols (Table 2). Gene therapy approaches aimed at correcting the defective gene directly in the patient’s hematopoietic stem cells may potentially avoid these risks by restoring osteoclast function without the need for complete bone marrow replacement and the associated immunosuppression.

### 4.2. Immunomodulatory Therapy

Interferon γ (IFN-γ) is a cytokine with immunomodulatory properties and the ability to enhance osteoclast activity through upregulation of RANKL expression and stimulation of bone resorption. This property has attracted attention to IFN-γ1b as a potential therapeutic agent for disorders characterized by impaired osteoclastic resorption, including osteopetrosis [34]. Administration of IFN-γ1b to patients with osteopetrosis significantly increased osteoclastic bone resorption, enhanced superoxide production in peripheral lymphocytes, and reduced infection rates [35]. In vitro studies using blood cultures from patients with osteopetrosis demonstrated that IFN-γ1b promotes osteoclast formation [36].

In 2000, the drug Actimmune (Interferon γ1b) was approved by the FDA to slow disease progression in patients with severe malignant osteopetrosis. In a phase III clinical trial, 15 patients with osteopetrosis received either Actimmune or control vitamin D. The time to disease progression was significantly prolonged in patients receiving Actimmune (165 days) compared with those receiving the control treatment (65 days). Evidence of increased bone resorption, enhanced bone marrow activity, and a reduction in the number of severe infections was observed [36].

In some patients, the drug improved markers of bone resorption and hematological parameters; however, the results were heterogeneous, and the therapy did not represent a curative treatment. In randomized studies involving patients with autosomal dominant osteopetrosis, IFN-γ1b did not significantly increase bone resorption and was associated with adverse effects, which limits its use outside the context of preparation for transplantation [37].

### 4.3. Gene Therapy and Experimental Treatment Strategies

The development of gene and gene-cell therapy is considered one of the most promising approaches for the treatment of *TCIRG1*-associated osteopetrosis, as these strategies aim to eliminate the primary genetic defect underlying the disease. In recent years, experimental studies involving the use of lentiviral vectors and genome editing technologies have been actively conducted, demonstrating the potential for restoration of osteoclast function in preclinical models and providing a rationale for transition to clinical application.

Lentiviral gene addition in CD34^+^ hematopoietic stem cells currently represents the most advanced and clinically realistic approach. Moscatelli et al. demonstrated efficient *TCIRG1* gene transfer using a lentiviral vector under the SFFV (Spleen Focus-Forming Virus) promoter, resulting in stable expression in osteoclast precursors and restoration of functional bone resorption in vitro, including normalization of Ca^2+^ release and CTX-I levels [37,38]. Importantly, subsequent studies confirmed that TCIRG1 overexpression does not impair cell viability or differentiation capacity, supporting its suitability for clinical translation [39]. In murine models, lentiviral modification achieved durable engraftment, stable gene expression, and reversal of the osteopetrotic phenotype in the majority of transplanted animals without evidence of clonal dominance, indicating an acceptable preclinical safety profile [40,41]. Nevertheless, this approach still requires myeloablative conditioning and carries risks of insertional mutagenesis, which remain key limitations for clinical application.

Induced pluripotent stem cell (iPSC)-based strategies represent an alternative autologous gene-cell platform. Patient-derived iPSCs carrying pathogenic *TCIRG1* variants c.1549G>A and c.2236C>T have been successfully reprogrammed and differentiated into osteoclasts, recapitulating the disease phenotype with impaired expression of key bone-resorbing enzymes such as cathepsin K (CTSK) and tartrate-resistant acid phosphatase (TRAP). Transgenic correction of *TCIRG1* in these cells restored osteoclast function and bone resorption capacity, providing an important proof-of-concept for autologous cell-based correction strategies [41]. However, iPSC-based approaches remain limited by complex manufacturing processes, concerns regarding genomic stability, and challenges in scalable clinical translation, making them currently less practical than hematopoietic stem cell-based gene addition.

These studies highlight the potential for autologous gene-cell therapy, in which a patient’s own hematopoietic cells can be genetically modified ex vivo and returned to the patient, potentially circumventing complications associated with allogeneic transplantation.

Genome-editing approaches, including base and prime editing, represent the most precise but the least developed therapeutic strategy. These technologies offer the theoretical advantage of correcting single-nucleotide *TCIRG1* variants without introducing double-strand DNA breaks, thereby potentially reducing the risk of off-target effects and genomic instability. However, at present, their application in *TCIRG1*-associated osteopetrosis remains strictly preclinical. While proof-of-concept success has been demonstrated in other monogenic disorders, their relevance for osteopetrosis is currently limited to a subset of pathogenic variants and has not yet been validated in disease-specific in vivo models. Therefore, base and prime editing should be considered long-term future perspectives rather than near-term clinical options.

From a comparative standpoint, lentiviral gene addition currently represents the most translatable and scalable strategy, whereas iPSC-based approaches remain at an intermediate experimental stage, and genome editing technologies represent the most precise but still highly experimental direction. Each approach faces distinct challenges: safety and conditioning requirements for lentiviral therapy, manufacturing complexity for iPSC-based strategies, and delivery, efficiency, and regulatory barriers for genome editing.

In summary, a comparative assessment of the three strategies reveals a clear trade-off between readiness and precision. Lentiviral gene addition in HSCs is the most clinically ready approach, supported by robust preclinical efficacy and scalability, yet remains constrained by conditioning-related toxicity and insertional mutagenesis risks. iPSC-based platforms offer an autologous alternative with proven phenotypic rescue, but their complexity and manufacturing hurdles limit near-term applicability. Genome editing, while theoretically superior for correcting specific mutations, is still at an exploratory stage without disease-relevant in vivo validation. Thus, current translational efforts should prioritize lentiviral gene addition, whereas iPSC and editing technologies represent sequential layers of future optimization rather than immediate clinical options.

Despite these advances, several unresolved challenges remain, including limited engraftment efficiency in the sclerotic bone marrow niche, the need for conditioning regimens across most approaches, and economic constraints inherent to ultra-rare disease therapy development. In this context, while gene addition approaches are closest to clinical implementation, genome editing technologies should be viewed as a potential future refinement rather than an immediate therapeutic alternative.

Prime- and base-editing approaches are still under active development and are not yet widely applicable in clinical practice. It must be taken into account that the number of patients carrying any specific pathogenic variant is extremely small (except of a few regions), which raises questions about the economic feasibility of developing variant-specific editing therapies. In contrast, a gene-addition strategy using lentiviral modification of hematopoietic stem cells could potentially address the underlying defect in a broader group of patients, making it a more practical and scalable therapeutic approach.

## 5. Conclusions

*TCIRG1*-associated osteopetrosis is a severe hereditary disorder that, without timely intervention, can lead to profound skeletal pathology, hematological abnormalities, neurological complications, and early mortality. Accordingly, early diagnosis and prompt initiation of therapy is critical for the prognosis and the prevention of severe outcomes. Key unresolved challenges include the optimal timing of HSCT and the inability to reverse already established neurological damage, which underscores the importance of early intervention. Currently, hematopoietic stem cell transplantation remains the only standard treatment for early-diagnosed cases, allowing restoration of osteoclast function and prevention of major disease complications.

At the same time, gene therapy is an actively developing field, including lentiviral delivery of a wild-type *TCIRG1* gene and emerging genome-editing approaches, which may serve as alternatives or adjuncts to HSCT by reducing the risks of immunological rejection and expanding therapeutic options. Future research priorities should focus on early diagnosis, the establishment of multicenter patient registries, and the initiation of clinical trials for gene- and gene-cell-based therapies. Despite significant progress in preclinical studies, further work is required to enhance the safety, efficacy, and stability of engraftment of genetically modified cells in order for gene therapy to be implemented in clinical practice and provide long-term correction of the disease. Overall, a forward-looking clinical perspective emphasizes the need for integrated strategies that combine timely HSCT with the development of safe and effective gene-based interventions.

## Figures and Tables

**Table 1:** Diagnostic and therapeutic workflow for *TCIRG1*-associated osteopetrosis.

Step	Stage	Main Actions
1	Clinical suspicion	Assessment of early symptoms: anemia, thrombocytopenia, hepatosplenomegaly, hypocalcemic seizures, visual impairment, skeletal abnormalities
2	Radiological evaluation	X-ray/CT imaging: osteosclerosis, Erlenmeyer flask deformity, metaphyseal widening, “sandwich” vertebrae
3	Laboratory assessment	CBC (pancytopenia), calcium levels, evaluation of extramedullary hematopoiesis
4	Genetic testing	Multigene panel (including *TCIRG1*), single-gene sequencing if indicated, CNV analysis, WES/WGS in complex cases
5	Differential diagnosis	Distinguish from CLCN7-, OSTM1-, TNFSF11-, TNFRSF11A-associated osteopetrosis forms which is essential for therapeutic decision-making
6	Pre-transplant evaluation	HLA typing, donor search, clinical assessment of transplant eligibility
7	Treatment	Allogeneic HSCT (standard of care); supportive therapy; consideration of experimental gene-based approaches

**Table 2:** Summary of allogeneic HSCT outcomes in *TCIRG1*-associated infantile osteopetrosis [21,22,23,24,25,26,27,28,29,30,31,32,33]. HSCT—hematopoietic stem cell transplantation; HLA—human leukocyte antigen; OS—overall survival; EFS—event-free survival; GVHD—graft-versus-host disease; CIBMTR—Center for International Blood and Marrow Transplant Research. Survival outcomes refer to published cohort data.

Parameter	Key Findings
Donor type	HLA-identical related: best outcomes; HLA-matched unrelated: alternative; Umbilical cord blood: slower engraftment, higher risk of graft failure
Conditioning regimen	Myeloablative: busulfan + fludarabine (most common); treosulfan + fludarabine + thiotepa (alternative for high-risk patients)
Survival outcomes	200-patient cohort: 88% 5-year relapse-free survival (HLA-identical), 80% 5-year progression-free survival (HLA-matched unrelated); CIBMTR data: 62% relapse-free survival (HLA-identical); 746-patient cohort: 3-year OS 69%, EFS 61%, graft failure ~21%; improved survival with early HSCT (<6 months) and experienced centers
Post-HSCT complications	Acute and chronic GVHD, infectious complications, graft failure or unstable graft function, veno-occlusive disease, pulmonary hypertension, interstitial pneumonitis, hypercalcemia
Long-term outcomes	Visual impairment (~70%), hearing impairment (~10%), delayed motor development; irreversible neurological damage if HSCT performed late.

## Data Availability

No new data were created or analyzed in this study. Data sharing is not applicable to this article.
